# Microfiltration Membranes Modified with Composition of Titanium Oxide and Silver Oxide by Magnetron Sputtering

**DOI:** 10.3390/polym13010141

**Published:** 2020-12-31

**Authors:** Joanna Kacprzyńska-Gołacka, Monika Łożyńska, Wioletta Barszcz, Sylwia Sowa, Piotr Wieciński, Ewa Woskowicz

**Affiliations:** 1 Łukasiewicz Research Networks—Institute for Sustainable Technology, 6/10 Pułaskiego St., 26-600 Radom, Poland; monika.lozynska@itee.lukasiewicz.gov.pl (M.Ł.); wioletta.barszcz@itee.lukasiewicz.gov.pl (W.B.); sylwia.sowa@itee.lukasiewicz.gov.pl (S.S.); ewa.woskowicz@itee.lukasiewicz.gov.pl (E.W.); 2 Faculty of Materials Science and Engineering, Warsaw University of Technology, 141 Woloska St., 02-507 Warsaw, Poland; piotr.wiecinski@gmail.com

**Keywords:** polyamide membranes, surface modification, magnetron sputtering, TiO_2_ + AgO coatings, low-pressure plasma, plasma treatment

## Abstract

In this work, the authors present the possibility of modification of polymer membranes by TiO_2_ + AgO coating created by the magnetron sputtering method. The two-component TiO_2_ + AgO coating can improve and shape new functional properties such as bactericidal and photocatalytic properties. The influence of magnetron power changes on the structure of the membrane was investigated as well. The structure and elemental composition of TiO_2_ + AgO coatings were analyzed using SEM and EDS technique. All deposited coatings caused a total inhibition of the growth of two investigated colonies of *Escherichia coli* and *Bacillus subtilis* on the surface. The photocatalytic properties for membranes covered with oxide coatings were tested under UV irradiation and visible light. The filtration result show that polymer membranes covered with two-component TiO_2_ + AgO coatings have a permeate flux similar to the non-coated membranes.

## 1. Introduction

Microfiltration (MF) processes conducted with polymer membranes play an increasingly important role in many areas of the industry [1,2]. Due to their advantages, such as simplicity and application flexibility, these techniques are used in a variety of technological processes. However, membrane filtration processes also have disadvantages. The specificity of this process creates good conditions for the formation and deposition of biofilm on the active surfaces of membranes (biofouling). This requires membranes to be cleaned or replaced more frequently leading to a reduction in filtration efficiency and an increase in filtration costs [3,4,5]. Membranes made from polymers in many cases can be easily exposed to biofouling [6]. Microbial biofilm can form in both cases: living or non-living surfaces and are prevalent in natural, industrial and hospital settings [7]. The deposition of components from the feed solution and the growth of bacteria on the surface and inside the pores of the membrane causes malfunctions of devices or the increase in material and energy consumption [8,9].

Previous results evidenced an efficient modification of polymeric membrane surface by magnetron sputtering of metal oxide coatings that have the potential to prevent biofilm growth on the surface of the membranes [10,11]. This technology enables the production of thin coatings on the surface of the membrane providing various functional properties, which can improve the efficiency of membrane filtration and reduce the operating costs. The most extensively studied new material solutions (including membranes) concern hydrophilic and self-cleaning properties, which are based on metal oxides e.g., TiO_2_ [12,13,14]. These coating are non-toxic and characterized by high thermal and chemical stability and resistance to unfavorable environmental factors. Photocatalytic properties also determine the wide area of application for TiO_2_ as a self-cleaning coatings enabling the degradation of organic and inorganic compounds. In addition, these types of coating are characterized by a low water contact angle [15]. Nevertheless, the TiO_2_ coating itself shows bactericidal activity only in the presence of UV radiation [16,17]. The state of the art shows that doping the TiO_2_ coating with various metals, such as Cu, Zn, Cr or Ag, can contribute to the improvement of its functional properties [18,19,20,21]. As observed in our previous work, the best solution is to dope titanium oxide (TiO_2_) with silver oxide (AgO), mainly due to the strong bactericidal activity of this material. The work carried out by the authors showed, that by using AgO coating for the modification of polymer membranes resulted in a 100% reduction of bacteria on its surface [11]. Moreover, it has been proved that the Ag doping expands the light absorption of TiO_2_ in the visible light region [22,23,24]. It should contribute to the improvement of the efficiency of the degradation of organic and inorganic compounds on the membrane surface.

The study investigated how changes in the technological parameters of the TiO_2_ + AgO coating deposition process influence the antibacterial effect as well as the structure and filtration efficiency of membranes. The application of magnetron sputtering technology is an innovative method, which generates functional thin films on the surface of the membrane. Herein, the authors have shown the possibility of using the magnetron sputtering technique to deposit of TiO_2_ + AgO coatings on the surface of polymer microfiltration membranes in order to obtain antibacterial and photocatalytic properties. The influence of magnetron power changes on the structure of the membrane was also investigated. Structure and morphology of native and modified membranes were characterized. The bactericidal and photocatalytic properties of modified membranes, while retaining their filtration properties were confirmed as well.

## 2. Materials and Methods

### 2.1. Coatings Deposition

The TiO_2_ + AgO coatings were deposited by reactive magnetron co-sputtering technology (MS-PVD) using a Standard 3 device produced by Ł-ITeE (Radom, Poland). The device was equipped with a two magnetron plasma sources located on the same wall in the chamber. Titanium and silver targets were used at the same time of the deposition process. The purity of both targets was 99.99%. The targets diameter was about 100 mm and the distance between the sample and the targets was 200 mm. The coatings were deposited with a reactive gas atmosphere composed of a mixture of 10% oxygen (99.9999% purity) and 90% argon (99.9999% purity). The TiO_2_ + AgO coatings were prepared without the negative potential substrate polarization. The power of Ti magnetron source was variable in the range 650–1000 W. The power of Ag magnetron source was variable in the range 2580 W. The time of the MS-PVD process was 30 s.

### 2.2. Structure and Elemental Composition Characterization

The Hitachi Su-8000 scanning electron microscope (SEM; Tokyo, Japan) equipped with an electron gun with cold field emission was using for structure characterization of prepared coatings. The very good resolution with a relatively low beam current in this type of electron source is beneficial for observing materials sensitive to the electron beam, such as the analyzed membranes. The secondary electron signal (SE) was used for material observation but there was not deposited a conductive layer on the sample. The elemental composition of the tested material was determined by the EDX method.

### 2.3. Bactericidal Properties

Antibacterial properties of membranes modified with TiO_2_ + AgO were examined for microorganisms that were representative of Gram-negative (*Escherichia coli*) and Gram-positive (*Bacillus subtilis*) bacteria. Before microbiological tests, the membranes were sterilized with a UV-C lamp for t = 30 min. Microbiological tests were carried out using a vacuum filtration kit. An inoculum of the used bacteria was prepared in a saline buffer (KH_2_PO_4_, Chempur) from a 24-hour culture at a concentration of 1.5–3.0 * 10^5^ CFU/cm^3^. The obtained suspension was further diluted using the serial dilution method to achieve a countable number of colonies on the membranes. From the prepared dilutions, 0.04 cm^3^ (for *Escherichia coli*) and 0.1 cm^3^ (for *Bacillus subtilis*) of the suspension were taken and transferred to a sterile 1000 cm^3^ phosphate buffer. Then 10 cm^3^ of such prepared suspension, which was prepared in this way was filtered through the membranes under a pressure of 500 mbar using vacuum filtration kit. The membranes were placed on the plates containing Luria Bertani (LB, VWR) growth medium and incubated at 37 °C for 24 h. After this time, the bacterial colonies that had grown on the membranes were counted. The reference sample in the research was the unmodified membrane. Results of antibacterial activity of the membranes were expressed as the percentage (%) reduction in the colony (CFU) counts.

### 2.4. Photocatalytic Properties

Photocatalytic properties of the membranes were evaluated on the degree of methylene blue degradation (0.1% *v/v*, Science Company) under the UV and visible light. Polyamide membranes unmodified and modified with two-component TiO_2_ + AgO coatings were placed in Petri dishes. A 20 cm^3^ volume was applied to their surface. Both UV-A lamp and daylight were used to study the effect of the type of UV radiation on the photocatalytic properties. After 8, 24, 48 and 72 h of UV and visible light irradiation, spectrophotometric measurements were made at 665 nm using a Hach DR 6000 spectrophotometer (Hach Company, Loveland, CO, USA). The tests were repeated three times for each tested sample. In order to investigate the effect of doping TiO_2_ coatings with AgO on the photocatalytic properties in visible light, one-component coatings were also tested according to the same methodology.

### 2.5. Filtration Properties

The permeate flux was determined by measuring the time required to filter deionized water (100 cm^3^) through the membrane (8 cm^2^) under defined transmembrane pressure (500 mbar). The deionized water was characterized by the conductivity and pH of 5.3 μS/cm and 6.5, respectively. For this purpose, the laboratory “dead-end” filtration set-up was used. The filtration properties of the membranes were evaluated based on the permeate flux (Equation (1)).

A “dead-end” vacuum system, operating at a pressure of 0.5 bar, was used to determine the permeate flux. The permeate flux (*J_p_*) was calculated from the time (*t*) taken to filter 100 cm^3^ (*V_p_*) of deionized water through a membrane with an area of 8 cm^2^ (*A*) in accordance with Equation (1).

(1) $$J_{P}\;=\;\frac{V_{p}}{A\cdot t}$$

## 3. Results and Discussion

### 3.1. Structure and Elemental Composition Characterization

**Figure 1:**
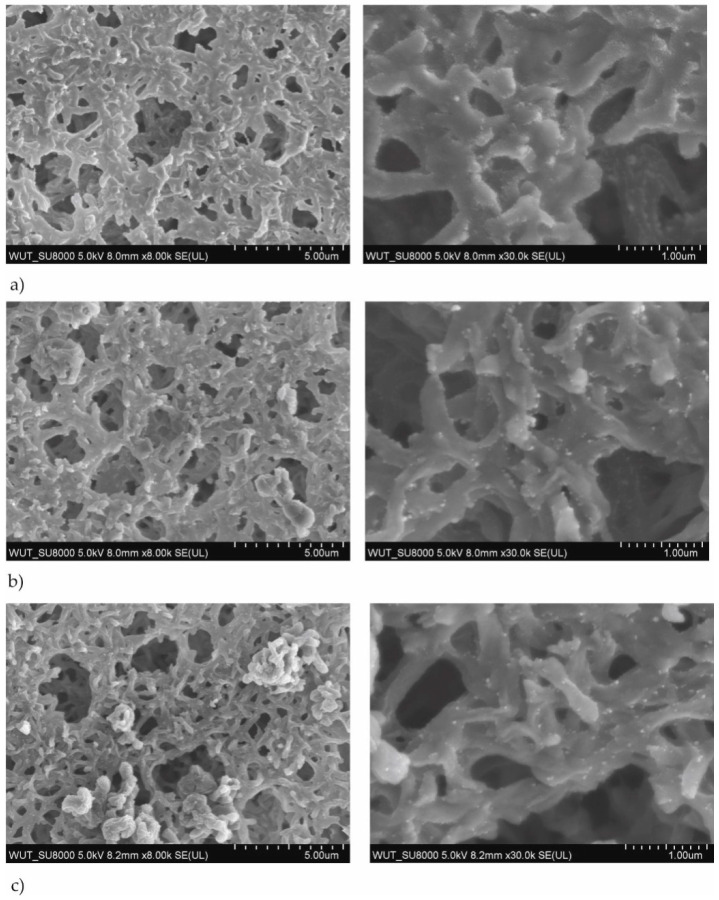
SEM images of the membranes with TiO_2_ + AgO coatings deposited at the different magnetron powers P_M_ (**a**) P_M-Ag_ = 80 W, P_M-Ti_ = 1000 W, t = 30 s, (**b**) P_M-Ag_ = 50 W, P_M-Ti_ = 1000 W, t = 30 s, (**c**) P_MAg_ = 25 W, P_M-Ti_ = 1000 W, t = 30 s.

**Figure 2:**
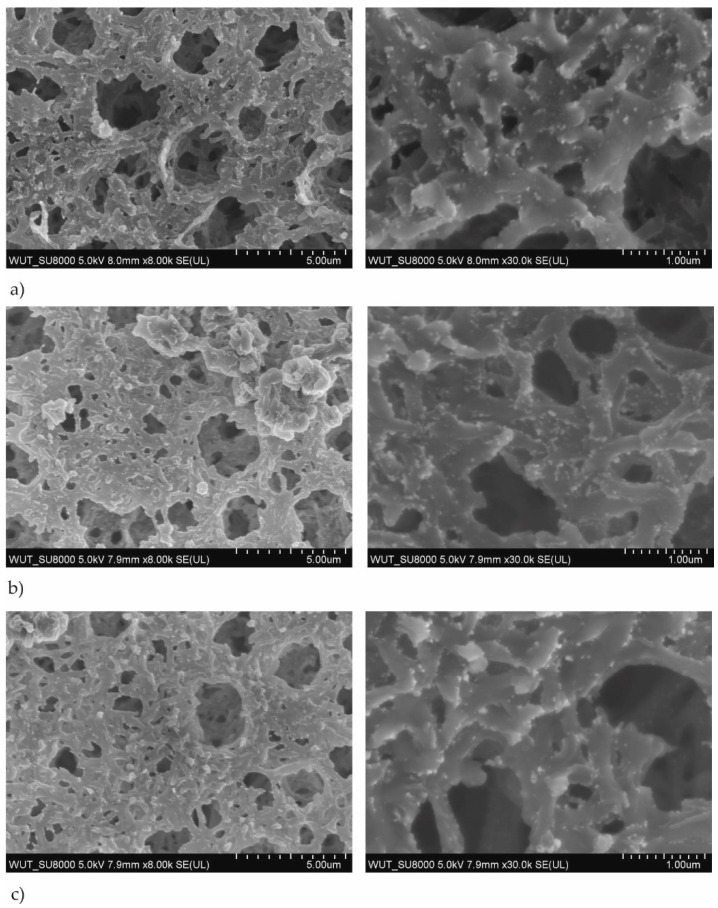
SEM images of the membranes with TiO_2_ + AgO coatings deposited at the different magnetron powers P_M_ (**a**) P_M-Ag_ = 80 W, P_M-Ti_ = 650 W, t = 30 s, (**b**) P_M-Ag_ = 50 W, P_M-Ti_ = 650 W, t = 30 s, (**c**) P_MAg_ = 25 W, P_M-Ti_ = 650 W, t = 30 s.

**Figure 3:**
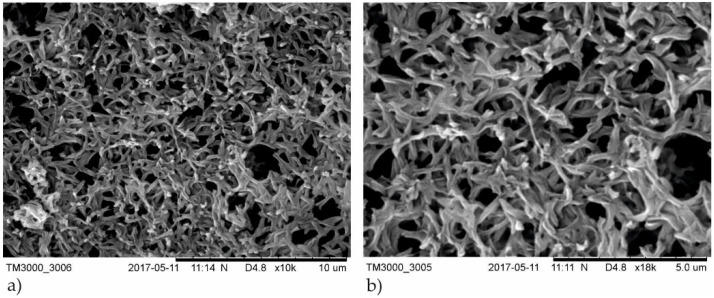
SEM images of the non-coated membranes: (**a**) magnification 10,000×, (**b**) magnification 18,000×.

The SEM images of the membranes coated with TiO_2_ + AgO were prepared for a different power of the magnetron source (P_M-Ag_ = 25 W, 50 W and 80 W, P_M-Ti_ = 650 W and 1000 W) used for modification were shown in Figure 1 and Figure 2. The SEM analysis of the structure of membrane with TiO_2_ + AgO coatings showed the comparison of the differences to non-coated membrane presented in Figure 3.

The SEM analysis showed that the structure of the membrane’s surface changed depending on the magnetron power. In the case of coatings created with the magnetron power P_MTi_ = 1000 W (Figure 1), the multiple bright AgO particles smaller than 100 nm were observed in their structure. The proportion of these particles decreased as the power P_M-Ag_ of the magnetron decreased. In the case of coatings created with smaller magnetron power P_M-Ti_ = 650 W (Figure 2), much fewer light particles were observed on the surface. The particles were localized mainly inside the pores, as presented in Figure 3. The correlation between the magnetron power and the number of AgO particles on the membrane surface has remained unclear.

**Figure 4:**
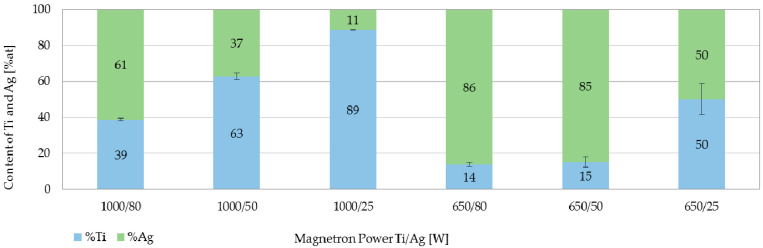
Elemental composition of TiO_2_ + AgO coatings deposited at different magnetron powers.

The elemental composition analysis included the assessment of the magnetron power effect on the percentage content for individual metallic elements in the elemental composition of the coating. The obtained results showed, that the TiO_2_ + AgO coatings (Figure 4) created at the higher magnetron power P_MTi_ = 1000 W were characterized by a higher content of Ti compared to the coatings deposited at the power of P_M-Ti_ = 650 W. The increase of the magnetron power P_M-Ag_ resulted in the increase of Ag content in the elemental composition of the tested coatings.

### 3.2. Antibacterial Properties

**Figure 5:**
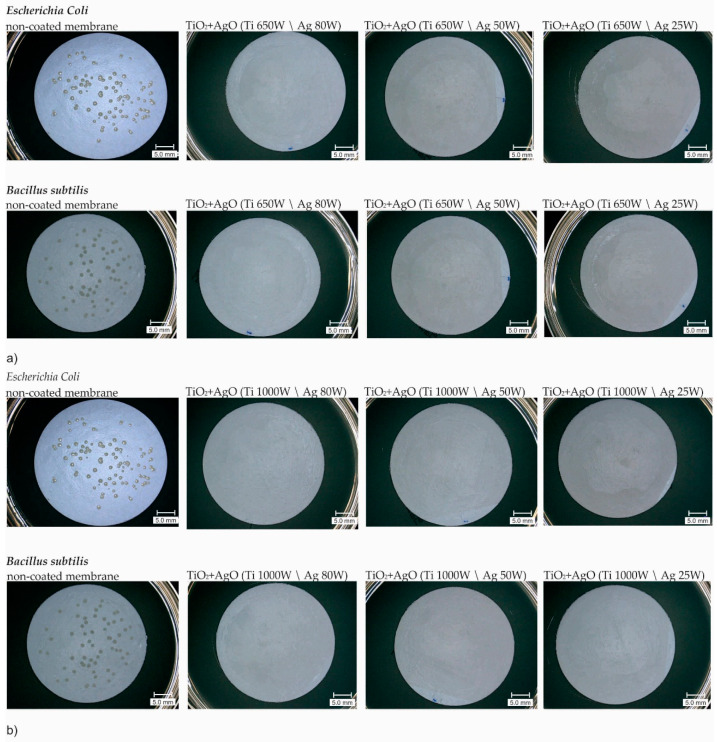
3D microscope images of TiO_2_ + AgO coated membranes after filtration of bacterial suspensions: (**a**) TiO_2_ + AgO modified membranes (P_M-Ag_ = 25 W, 50 W and 80 W; P_M-Ti_ = 650 W); (**b**) TiO_2_ + AgO modified membranes (P_M-Ag_ = 25 W, 50 W and 80 W; P_M-Ti_ = 1000 W).

The antibacterial activity of membranes modified by TiO_2_ + AgO coatings was specified against two representative bacteria such as *Escherichia coli (E.coli)* and *Bacillus subtilis (B.subtilis)*, respectively. The coatings generated with the following power of magnetron source (P_M-Ag_ = 25 W, 50 W and 80 W, P_MTi_ = 650 W and 1000 W) lead to the complete growth inhibition of the colonies of *E.coli* and *B.subtilis* on the membranes which is shown in Figure 5. Both bacteria showed no growth on the agar plates after incubation.

**Figure 6:**
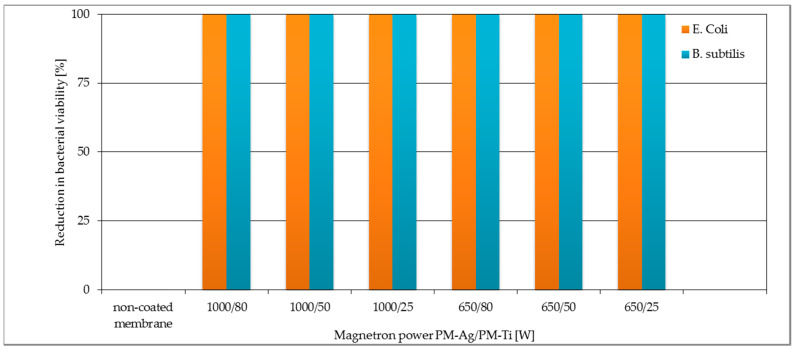
Reduction [%] CFU counts of *Escherichia coli* and *Bacillus subtilis* on no-coated membrane and membranes covered with TiO_2_ + AgO coating deposited during 30 s at different magnetron powers P_M-Ti_ and P_M-Ag._

These results were similar to those obtained in the previous study, where the AgO coated membranes with different power of magnetron source caused total growth inhibition of *E. coli* and *B. subtilis* [11]. The results of reduction (%) in CFU counts are presented in Figure 6. For each TiO_2_ + AgO modified membranes the antibacterial activity of the membrane amounted to 100% compared to the no-coated membrane.

The obtained results may be associated with the elemental composition of the coatings, which created with MS-PVD technique. The deposited coatings on the polymer membrane were in the form of a solid titanium oxide matrix where Ag/AgO nanoparticles were embedded, (Figure 2 and Figure 3). The bactericidal properties of the TiO_2_ + AgO composite coating were probably related to the presence of silver/silver oxide (Ag/AgO) nanoparticles in this coatings. Research by Thukkaram et al. confirmed that the enrichment of the titanium oxide coating with silver nanoparticles increases the antimicrobial activity against Gram-positive and Gram-negative bacteria [25]. Navabpour et al. proved that TiO_2_ + AgO coatings formed by using the reactive magnetron sputtering have stronger bactericidal properties than TiO_2_ coatings produced in the same ways [26]. The strong bactericidal properties of silver/silver oxide (Ag/AgO) nanoparticles were confirmed in many studies [27,28,29]. Silver and silver oxide nanoparticles have an affinity for functional groups included in cellular proteins and nucleic acids. The binding silver ions in the cytoplasmic membrane of the bacterial cell cause their accumulation and its destabilization and increase in membrane permeability. As a consequence, this leads to the uncontrolled transport of protons and depolarization of cell membrane, and death of microorganisms [30]. The antibacterial activity of silver and silver oxide nanoparticles can be associated with free radicals, which they generated. The free radicals cause oxidative stress in bacterial cells. Li et al. compared the relationship between the antimicrobial activity of nanoparticles of various metal oxides and oxidative stress. The viability of *E.coli* cells in solutions of metal oxide nanoparticles under the influence of UV radiation was reduced [31].

### 3.3. Photocatalytic Properties

**Figure 7:**
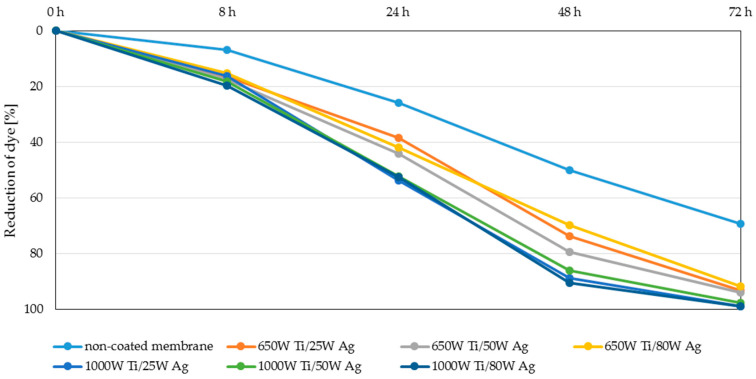
Reduction of the MB dye under the influence of UV-A light.

**Figure 8:**
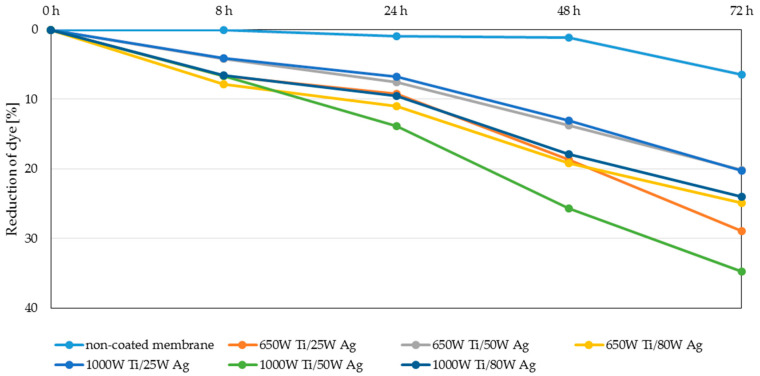
Reduction of the MB dye under the influence of visible light.

The membranes with two-component coatings were subjected to photocatalytic tests. The results obtained for TiO_2_ + AgO coatings after irradiation with a UV lamp and daylight are presented in Figure 7 and Figure 8, respectively.

As shown in Figure 7 the membranes with TiO_2_ + AgO coatings deposited with the magnetron power P_M-Ti_ = 1000 W showed better photocatalytic properties after irradiation UV lamp than the coatings deposited with the magnetron power P_M-Ti_ = 650 W (Figure 7). For all types of coatings with P_M-Ti_ = 1000 W over 90% reduction of the dye was achieved after 72 h. The literature and own research data show that titanium oxide is a substantial photocatalytic factor in UV light [32,33]. The doping of the coatings with silver oxide (AgO) for bactericidal and photocatalytic properties in visible light does not negatively influence photocatalytic properties under UV light.

**Figure 9:**
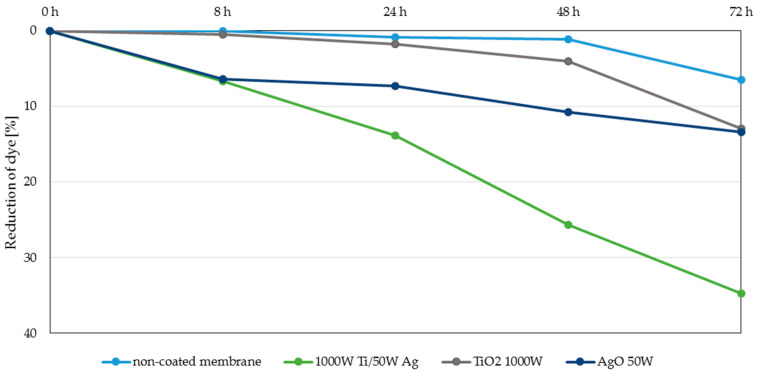
Reduction of the MB dye under the influence of visible light.

The highest degree of dye reduction was achieved for membranes with magnetron power 1000 W Ti/50 W Ag and coatings with magnetron power 650 W Ti/25 W Ag (35 and 28% respectively) can be observed when two-component coatings are exposed to daylight. The achieved degree of dye reduction was much lower than that of UV irradiation. This is due to the fact that both TiO_2_ and AgO do not have photocatalytic properties in visible light. Normally, with light irradiation UV for titanium oxide, an electron from the valence band can be promoted to the conduction band, leaving an electron. This deficiency is known as a hole in the valence band and is causing an excess of negative charge in the conduction band. These generated species can participate in surface redox reactions and generate secondary reactive oxygen species. However, the excited reactive electron is unstable, and it can return to the valence band, to be stable again, provoking recombination. Studies revealed, that doping of silver improves the photocatalytic activity of TiO_2_ inducing an efficient surface plasmon resonance effect under sunlight. That prevents the recombination of e^−^-h^+^ pairs, which is responsible for the decreasing process of the photocatalytic activity of TiO_2_ [34,35,36,37,38]. In order to assess the effect of doping titanium oxide coating with silver oxide, additional experiments were conducted in visible light for one-component coatings. The results are presented in Figure 9.

The obtained results showed that the tested one-component TiO_2_ coating and AgO coatings have lower photocatalytic activity and yielded an approx. 10% reduction of the dye after 72 h. Doping the titanium oxide coating with silver oxide caused an even more than threefold increase in the photocatalytic properties of these membranes. The catalytic properties of such prepared membranes are influenced by many factors, including the quantity and size of the silver particles [35,36], which was confirmed during this study. In the case of the tested two-component coatings, the highest dye reduction was obtained for coatings with a silver content between 50 and 60% (Figure 2). The decomposition of MB dye in visible light was not as intense for the coating with the highest Ag content (90% for 1000 W Ti/80 W Ag coatings) or the lowest one (about 15% for 650 W Ti/50 W Ag and 650 W Ti/80 W coatings).

### 3.4. Filtration Properties and Stability of the Coatings

**Figure 10:**
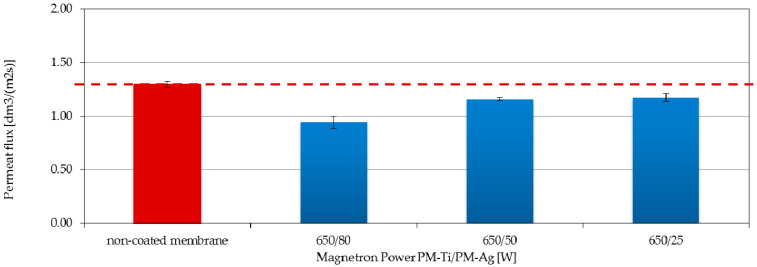
Effect of the P_M-Ag_ magnetron power on the permeate flux of membranes covered with TiO_2_ + AgO coatings deposited at a constant power P_M-Ti_ = 650 W.

**Figure 11:**
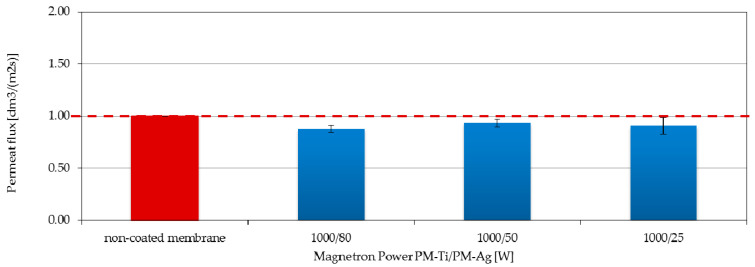
Effect of the P_M-Ag_ magnetron power on the permeate flux of membranes covered with TiO_2_ + AgO coatings deposited at a constant power P_M-Ti_ = 1000 W.

The results of the filtration and transport properties analysis for polymer membranes coated with two-component TiO_2_ + AgO coatings showed an inconsiderable decrease in the permeate flux compared to the native membrane (Figure 10 and Figure 11). The highest (27%) decrease in the permeate flux compared to the native membrane was recorded (Figure 10) in the case of the membrane with the coating deposited at the magnetron powers P_M-Ti_ = 650 W and P_M-Ag_ = 80 W. Reducing the power of the P_M-Ag_ magnetron resulted in an improvement of the permeate flux. After increasing the magnetron power to P_M-Ti_ = 1000 W, a significant improvement in the filtration properties was observed. The permeate flux was comparable to the non-coated membrane (Figure 11) for the membrane with the coating deposited at the magnetron powers P_M-Ag_ = 25 W and P_MAg_ = 50 W.

The increasing of the magnetron power P_M-Ti_ caused the increase of the proportion of hydrophilic TiO_2_ [15] in the coating structure and it can improve the hydrophilic properties of the whole coating, and consequently also the membrane surface. As a consequence, the filtration performance of the membrane will be most likely improved. The opposite situation was observed when the power P_M-Ag_ was increased. It caused an increase in the proportion of hydrophobic AgO in the coating structure. This can lead to a reduction in the hydrophilic properties of the membrane surface, which results in a decrease in its filtration properties.

## 4. Conclusions

In this study, the importance of an interdisciplinary approach was highlighted towards novel trends in the development of materials based on polymer membranes. Using the MS-PVD method can provide new functional properties for polymeric membranes. In the article, the different magnetron powers of Ti and Ag were used to create two-component coatings with new, common properties. Based on the surface analysis of the membrane the presence of AgO nanoparticles were observed. The size of these particles was smaller than 100 nm for magnetron power P_M-Ti_ = 1000 W. Research has shown that the magnetron power has effects on the percentage content for individual metallic elemental composition. The presence of AgO nanoparticles was related to the island nature of the AgO coating grown on a polymer substrate [11]. The antibacterial properties of TiO_2_ + AgO coatings were determined for the two following bacteria: *Escherichia coli* and *Bacillus subtilis*. The TiO_2_ + AgO coatings deposited at different magnetron powers resulted in complete reduction of growth of two representative bacteria. It was related to the presence of Ag/AgO nanoparticles in the coating structure, which exhibits strong antibacterial properties. All the tested membranes with TiO_2_ + AgO coatings indicated very good photocatalytic properties when irradiated with UV. The doping of TiO_2_ coating with AgO led to an increase in photocatalytic properties in visible light compared to the one-component coating on the polymeric membranes. At the same time, the authors reported no negative influence of the coating on the filtration properties of the membrane. In the case of the membrane with the TiO_2_ + AgO coating deposited on the membrane at the magnetron powers, P_M-Ti_ = 1000 W the permeate flux was similar to the no-coated membrane. The achieved results are very promising for polymeric material science, giving a prospect for potential application of thin film TiO_2_ + AgO coatings onto the surface of polyamide membranes.

## Data Availability

Not applicable.
